# mSphere of Influence: Pertussis Vaccination and Antibodies in Mothers and Infants

**DOI:** 10.1128/msphere.00009-23

**Published:** 2023-02-02

**Authors:** Susana Portillo

**Affiliations:** a Center for Vaccine Development and Global Health, University of Maryland School of Medicine, Baltimore, Maryland, USA; b Department of Pediatrics, University of Maryland School of Medicine, Baltimore, Maryland, USA

**Keywords:** pertussis vaccine, maternal vaccine, Tdap, infant immunity, antibody titer

## Abstract

Susana Portillo works in the field of mother-infant immunity with an emphasis on vaccination and prevention of respiratory diseases. In this mSphere of Influence, she reflects here on how two pertussis vaccine articles made an impact on her research. She discusses how much more remains to be understood about the role of maternal antibodies in preventing or reducing infant illnesses, their capacity to engage other immune components to deliver an efficient antimicrobial response, and their influence on the infant’s own response to vaccination. She emphasizes the need for safe and effective interventions that strengthen maternal and infant immunity before and after birth.

## COMMENTARY

Vaccines are among the most successful public health interventions that have improved quality of life by reducing or eliminating serious infectious threats. The effectiveness of vaccine intervention stands on its capacity to prevent or reduce risk of illness. As the science developed, vaccine-induced immunity has been inferred on the evidence of specific antibodies. Considering the diversity of the antibody repertoire and impact on function, the magnitude of vaccine-specific antibodies portrays an incomplete picture. Probing into how antibodies protect us from disease and for how long and answering associated questions are important to understanding vaccine performance and population immunity and are vital to the overarching goal of controlling and preventing diseases. However, detailed inquiries about what the vaccine-specific antibodies are doing, exactly how they work, and how long they last are scarce in the literature. The articles by Healy et al., “Kinetics of maternal pertussis-specific antibodies in infants of mothers vaccinated with tetanus, diphtheria and acellular pertussis (Tdap) during pregnancy,” (1) and Havers et al., “Maternal tetanus toxoid, reduced diphtheria toxoid, and acellular pertussis vaccination during pregnancy: impact on infant anti-pertussis antibody concentrations by maternal pertussis priming series,” (2) prompted me to think about antibodies beyond just a titer (an amount) and instead led me to consider their overall relevance, mechanistic effect, and contribution to preventing diseases in the mother-infant dyad.

Maternal vaccination augments the immunity naturally passed to the fetus and infant through the placenta and breast milk. The nursing infant receives ample immunity from the mother in this manner, which is critical to prevent infection in the first months of life until the child’s own immunity develops following routine immunization. Currently, influenza and Tdap vaccines are given during pregnancy, and the more recent coronavirus disease 2019 (COVID-19) vaccine is now also recommended. Through such vaccination efforts, infant morbidity and mortality have declined dramatically since the administration of the combined pertussis vaccine in 1948 (3). Despite this success, there are many aspects of host immunity related to maternal vaccination that need to be elucidated to help it achieve its utmost potential.

Both papers mentioned above point out two important outcomes of mother-infant pertussis immunization that require particular attention to maximize the benefit to the vulnerable infant population. First, Healy determined the antibody content in both the mother and the infant and described a faster decline in infant circulating IgG antibodies from what was previously reported (4). Healy’s study involved a kinetics and half-life interrogation of maternal pertussis-specific IgG induced by Tdap vaccination in a cohort of 34 healthy mother-infant pairs. The group found that placental transport of pertussis-specific IgG was efficient, but antibodies in the infant waned quickly in just over 4 weeks, at least ~2 weeks quicker than was previously thought (4). Healy and colleagues assessed serum IgG antibody levels against pertussis virulence factors pertussis toxin (PT), filamentous hemagglutinin (FHA), pertactin (PRN), and fimbriae (FIM) and recorded half-lives of 29.4 days for PT, 29.8 days for FHA, 31.2 days for PRN, and 35.8 days for FIM. This is an important finding with public health implications, since infants do not receive their primary vaccination series until 2 months of age in the United States and Europe; these results suggest that infants would be lacking protective immunity for a few months.

Second, Havers described differences in antibody levels among women who received whole-cell (wP) vaccine in their childhood vaccine series compared to those who received the acellular (aP) pertussis vaccine after the 1997 Advisory Committee on Immunization Practices (ACIP) recommendations. Different combination vaccines have been used to prevent whooping cough in various populations, including Tdap and DTaP, which contain acellular pertussis antigens (PT, FHA, and PRN), and DTwP, a whole-cell pertussis vaccine that contains PT, FHA, PRN, and also FIM; all such vaccines also contain diphtheria (DT) and tetanus (TT) toxoids. Combination vaccines that include DTaP plus hepatitis B, polio, or Haemophilus influenzae type B are also available for children. The Havers study examined prevalence of immunity based on type of vaccine received. The antibody level difference resulting from wP and aP priming was assessed in a cohort of 1,012 infants divided into the following three groups: those born to mothers who received aP in their primary childhood vaccine series (aP primed), those born to mothers who received whole-cell vaccine (wP primed), and infants from mothers primed with either vaccine (mixed wP/aP primed). The serological analysis in this study was focused on antibodies to PT. While there is no firm correlate of protection for pertussis, PT-specific IgG are necessary to prevent disease. Some have argued that PT IgG levels above 5 IU/mL could be considered to be protective (5); others have suggested a higher and more conservative cutoff level of 10 IU/mL (6), and more recently, 20 IU/mL has also been proposed (7, 8). The analysis by Havers et al. showed that the mean anti-PT IgG level in cord blood in the aP-primed group was 17.3 IU/mL, which was significantly lower (less than half) than the mean levels detected in the wP-primed (36.4 IU/mL) and mixed (31.2 IU/mL) groups. The ratio of placental PT antibody transfer was also lower in the aP-primed group (1.38) than in the wP-primed group (1.50) and mixed group (1.42).

The difference in circulating PT IgG levels in aP-primed childbearing-age women and their offspring is a paramount finding, as lower availability of PT antibody might compromise protection in the mother-infant dyad, with the infant being more susceptible and at higher risk of infection. While the overall implications of these findings are unclear, they warrant corroboration and careful examination, as they could have a major impact on public health. Currently, in winter 2023, there is a surge in respiratory syncytial virus (RSV), influenza, and COVID-19 in young children that has overwhelmed US health care facilities (9). Efforts should be undertaken to prevent resurgence of another potentially devastating childhood health threat, like pertussis.

As maternal vaccination continues to be refined and adopted by other countries, finding the best strategy to make the most of maternal pertussis immunization is essential to reducing disease in infants. The pertussis incidence in infants of <1 year of age continues to be the highest reported rate among different age groups; cases of pertussis began gradually increasing in the 1980s, reaching over 18,000 cases reported worldwide in 2019 (3). In addition, a deeper understanding of the structure, quality, and function of antibodies produced by pertussis and other vaccines administered during pregnancy and transferred to the infant and how these antibodies engage with the infants’ own immune systems would allow us to identify the most effective strategies to maximize antibody-mediated antimicrobial function and protection from infectious threats.

Without a firm correlate of protection against pertussis, it is difficult to know exactly what an antibody titer means for the mother or infant at the time immunity is measured. Vaccine efficacy can be surmised from epidemiological data as cases decrease over time, but there is a critical gap in knowledge regarding protective thresholds and basic functions of these vaccine-induced antibodies and mechanisms by which they act. Together, these two papers have helped me appreciate the knowledge gaps and strategize my own studies and future interests by focusing not only on the kinetics but also on functional aspects of antibodies in the mother-infant dyad. For example, I am presently applying cutting edge confocal imaging and data analysis that uses artificial intelligence technology to establish a high-throughput functional assay for quantification of antibody-mediated PT neutralization.

These two articles helped me realize the significance and complexity of mother-infant immunity and the importance of understanding the factors that influence its origin, persistence, and function as well as the importance of means to monitor and improve mother-child immunization. These concepts have driven my interest in diving deeper to understand what the antibodies are doing, how they do it, and how to achieve the best antibody responses in the context of maternal immunization as I seek to uncover new biology rules and generate new insights for future vaccine development endpoints and assessments.

There is an opportunity to tackle pertussis and other immunization challenges through well-characterized human studies, including ongoing controlled infection models, and by making use of technological advances. For example, the Bordetella pertussis community has been actively looking to expand and finesse the pertussis serological assays that are commonly used but not yet harmonized across laboratories. Currently, antibody titers are measured by either enzyme-linked immunosorbent assay (ELISA), Luminex, or Meso Scale Discovery (MSD) assays and calibrated against the WHO First International Standard Pertussis Antiserum 06/140. Functional assays, such as B. pertussis opsonophagocytosis and PT neutralization, hold promising value to evaluate antibody functional activity; development and optimization efforts are under way. Furthermore, breast milk, a rich resource for infant immunity, has yet to be explored for its full protective potential, including the presence in breast milk of functionally active antibodies, an important focus of my own research. Recently, various groups have used the systems serology platform to characterize maternal antibody transfer, focusing on various pathogens such as HIV, *Mycobacterium tuberculosis*, and Ebola virus and more recently severe acute respiratory syndrome coronavirus 2 (SARS-CoV-2) and *Shigella* (10–13). This approach has also been applied to maternal pertussis vaccination (14). This elegant tool can open up new avenues to help investigators analyze longitudinally the functional and biochemical characteristics of the antibody repertoire elicited by vaccine candidates for the mother-infant dyad that are crucial to reaching global public health goals.
